# Comparative Microbial Analysis of Paired Amniotic Fluid and Cord Blood from Pregnancies Complicated by Preterm Birth and Early-Onset Neonatal Sepsis

**DOI:** 10.1371/journal.pone.0056131

**Published:** 2013-02-20

**Authors:** Xiaowei Wang, Catalin S. Buhimschi, Stephanie Temoin, Vineet Bhandari, Yiping W. Han, Irina A. Buhimschi

**Affiliations:** 1 Department of Periodontics, Case Western Reserve University, Cleveland, Ohio, United States of America; 2 Department of Obstetrics, Gynecology and Reproductive Sciences, Yale University, New Haven, Connecticut, United States of America; 3 Department of Pediatrics, Yale University, New Haven, Connecticut, United States of America; 4 Department of Pathology, Case Western Reserve University, Cleveland, Ohio, United States of America; 5 Department of Reproductive Biology, Case Western Reserve University, Cleveland, Ohio, United States of America; Columbia University, United States of America

## Abstract

**Background:**

16S rRNA-based genomic analyses have revolutionized our understanding of infectious diseases. Many cases which were recognized as “idiopathic” are now known to have an infectious etiology. Here, we present a proof-of-concept study to examine the microbial link between intra-amniotic infection (IAI) and early-onset neonatal sepsis (EONS).

**Results:**

Using culture independent methods, we analyzed paired amniotic fluid (AF) and cord blood (CB) samples from 36 singleton pregnancies complicated by preterm birth (PTB), IAI, and/or EONS. PTB cases were grouped as **1)** Group 1– neonatal blood culture-positive EONS (n = 6). **2)** Group 2– neonatal blood culture-negative presumed EONS with positive IAI (n = 16). **3)** Group 3– neonatal blood culture-negative presumed EONS with no IAI (n = 7); **4)** Group 4– no EONS or IAI (n = 7). In addition, samples from term healthy deliveries (n = 8) served as technical controls. A total of 31 species (15 non-redundant) were identified in AF, of which only 1/3 were cultivated. Significantly fewer microorganisms were detected in CB, with a total of 18 species (7 non-redundant) identified, of which only 2 (*Escherichia coli*, *Streptococcus agalactiae*) were cultivated. Of those, *Bergeyella, Fusobacterium nucleatum*, and *Sneathia sanguinegens* had not been detected in EONS before. The novel species identified in AF by PCR include *Peptoniphilus harei* and *Lachnospiraceae* sp. The majority (72%) of CB species were also detected in the matching AF, with *E. coli* and *F. nucleatum* as the most prevalent. The 16S rRNA sequences of paired AF and CB were 99.9–100% identical, while no identical sequences were found between different pregnancies.

**Conclusions:**

Previously unrecognized, uncultivated or difficult-to-cultivate species are implicated in EONS. Microbial species in paired AF and CB likely share the same infectious origin. Given its prevalence in EONS, *F. nucleatum* should be placed on the same importance scale as *E. coli*.

## Introduction

Studies of microbes have traditionally focused on bacterial species isolated in culture. Because a large proportion of bacteria are uncultivated, traditional microbiological cultures provide an incomplete picture of the human microbiome [1]. Metagenomic techniques and sequencing technology have revolutionized our understanding of the bacterial diversity [2]. Sequencing of the small-subunit ribosomal RNA (16S rRNA) gene, which is highly conserved among all prokaryotes, yet is variable enough to allow differentiation of species [3], [4], has made it possible to identify microbial species that cannot be grown in culture or recognized based on phenotypic properties alone [5].

We were among the first to employ metagenomic approaches to evaluate amniotic fluid (AF) microbial diversity in pregnancies complicated by preterm birth (PTB) [6], [7]. Applications of 16S rRNA-gene sequencing suggested the existence of a far greater microbial diversity in AF than appreciated based on culture-dependent methods. In pregnancies where there was evidence of an inflammatory host response, approximately two thirds of microbes detected by culture-independent methods were not isolated by cultures. These included both uncultivated and difficult-to-cultivate species, such as *Fusobacterium nucleatum*, *Leptotrichia* (*Sneathia*), *Bergeyella*, *Peptostreptococcus*, *Bacteroides* and a species of the order *Clostridiales*. Interestingly, women with positive microbial cultures and/or PCR results often delivered neonates with early-onset neonatal sepsis (EONS) [7].

EONS (sepsis in the first 3 days of life) remains a major cause of neonatal morbidity and mortality [8]. Based on microbial cultures alone, the most frequently responsible pathogens are *Streptococcus agalactiae* (also known as Group B *Streptococcus*, or GBS) and *Escherichia coli* [8]. However, clinical manifestations of EONS (lethargy, apnea, respiratory distress, hypoperfusion and shock) often occur in newborns despite negative microbial cultures. In such clinical circumstance the customary diagnosis is “presumed” EONS. This practice denotes etiologic and pathogenic uncertainty with respect to origin of clinical symptoms and underscores that anti-microbial treatment during the first days of neonatal life cannot be reliant on microbiological test results. Plausible explanations as why a diagnosis of “confirmed” EONS is challenging are the narrow spectrum of pathogens identifiable by conventional microbiologic techniques and the widespread maternal use of antenatal antibiotic prophylaxis [9], [10].

The current view is that EONS is a syndrome with at least 3 pathogenic variants [10], [11]. In its first variant (EONS-I), microbial pathogens translocate from AF cavity to the fetus. Similar to intra-amniotic infection (IAI), it is conceivable that fastidious and uncultivated bacteria play a role [7]. If true, this may explain why a heightened inflammatory state is present at birth in many preterm newborns where traditional microbial cultures of AF and neonatal blood remain negative. Alternatively, it would suffice for endotoxin, other pathogen-associated molecular patterns (PAMPs), damage associated molecular patterns (DAMPs) (EONS-II), or just cytokines (EONS-III) to translocate from AF into the fetal circulation to elicit a fetal inflammatory process and induce clinical manifestations consistent with EONS [12], [13], [14].

Metagenomics has played a pivotal role in characterizing pathogenic microbiota and is becoming the new “gold standard” for the diagnosis of infectious diseases [6], [7], [15], [16], [17], [18]. We hypothesized that uncultivated and difficult-to-cultivate organisms in AF carry the potential to breach the fetal barrier, translocating to CB of neonates causing EONS (EONS-I). To test this hypothesis we examined the presence of microbial species in paired AF and CB in pregnancies complicated by IAI and/or EONS using culture-dependent and independent methods.

## Materials And Methods

### Patients And Study Groups

We studied paired AF-CB samples retrieved from 44 women pregnant with singletons. The research protocol was approved by the Human Research Protection Program Review Board at Yale University and Internal Review Board at Case Western Reserve University. All patients provided written informed consent.

Biological samples originated from a cohort of consecutive patients enrolled at Yale-New Haven Hospital (YNHH) from September 2004 to February 2009 who had paired AF and CB available for analysis (n = 161). To avoid selection bias, cases fulfilling the clinical group requirements were selected consecutively based on the availability of at least 1 ml umbilical vein CB for DNA extraction and other research analyses. The study groups were designed using the results of AF and neonatal blood bacterial cultures as reported by YNHH microbiology laboratory. The following groups were studied: **Group 1:** premature newborns with neonatal blood culture “confirmed” EONS (gestational age [GA] at delivery, median [interquartile range]): 25 [25]–[26] weeks, n = 6). **Group 2:** premature newborns with negative neonatal blood culture but “presumed” EONS and positive IAI (GA: 27 [25]–[29] weeks, n = 16). **Group 3:** premature newborns with negative neonatal blood culture but “presumed” EONS and negative IAI (GA: 32 [31]–[33] weeks, n = 7). **Group 4:** premature newborns without EONS and no IAI (GA: 32 [31]–[34] weeks, n = 7); **Group 5:** term healthy newborns (GA: 39 [38]–[39] weeks, n = 8) delivered by elective Cesarean within the same time period. This last group served as technical control for handling and analysis of the samples.

Gestational age was established based on the last menstrual period and/or ultrasound evaluation. Term delivery was defined as birth at GA ≥37 weeks. Preterm labor was defined as presence of regular uterine contractions, advanced cervical dilatation (≥3 centimeters) or effacement at GA<37 weeks [19]. No fetus exhibited evidence of anatomic structural abnormalities. Maternal medical conditions such as hypertension, preeclampsia, diabetes, thyroid disease, cholestasis, connective tissue diseases, viral infections (human immunodeficiency virus, hepatitis B, hepatitis C), fetal intrauterine growth restriction (estimated fetal weight <10^th^ percentile), and anhydramnios were excluded. A diagnosis of preterm premature rupture of the membranes (PPROM) was established following clinical evaluation of the patient by sterile speculum exam, complemented by a “nitrazine”, “ferning”, and “amnio-dye” tests [7]. Clinical chorioamnionitis was established in the setting of maternal fever (>37.8°C), maternal leukocytosis (≥15,000 cells/mm^3^), trans-abdominal uterine tenderness and/or foul smelling AF [14].

### Clinical Management Of The Patients

All mothers of the 36 premature newborns in **Groups 1–4** presented with symptoms of preterm labor or PPROM had amniocentesis to rule-out IAI. Following amniocentesis, each woman was followed prospectively till delivery. Corticosteroids for lung maturity were administrated if patients were ≤34 weeks GA or <32 weeks for PPROM cases. Digital exams were not permitted, unless clinically indicated. Antibiotic prophylaxis was indicated for extending latency period or for GBS as appropriate and in accordance to the American College of Obstetrics and Gynecology [19], [20], [21]. PPROM patients were given a combination of initial intravenous therapy (48 hours) with ampicillin (2-g dose every 6 hours) and erythromycin (250-mg dose every 6 hours), followed by oral therapy of 5 days with amoxicillin (250-mg dose every 8 hours) and enteric-coated erythromycin base (333-mg dose every 8 hours) if prior to 34 weeks of gestation [20]. All women in whom recto-vaginal cultures were positive for GBS received intrapartum antibiotic prophylaxis (penicillin, cefazolin, clindamycin, erythromycin, vancomycin as appropriate) in labor unless a cesarean delivery was performed before onset of labor in a woman with intact amniotic membranes [22]. Patients who delivered by Cesarean section received perioperative antibiotics, before skin incision [21]. In such cases prophylaxis consisting of a single dose of first-generation cephalosporin (i.e. cefazolin, 1 gram, intra-venous) was administered within 60 minutes before the start of the surgery, unless patient received antibiotics for the treatment of chorioamnionitis [21]. If the amniocentesis results were suggestive of infection, delivery of the fetus was indicated. In the setting of negative amniocentesis results, the patients were managed expectantly and monitored for signs and symptoms of clinical chorioamnionitis until delivery. Patients in **Group 5** were healthy women without any significant past medical history undergoing planned Cesarean delivery in the absence of labor. Indications for abdominal delivery included prior uterine surgery or breech presentation. All infants were appropriately grown for GA and had reassuring fetal heart rate patterns prior to surgery. The decisions for amniocentesis or indicated delivery including Cesarean were made independent of the research protocol.

### Clinical Management Of The Newborns And Diagnosis Of Eons

All premature neonates (**Groups 1–4**) were admitted in YNHH Neonatal Intensive Care Unit (NICU) and underwent clinical and laboratory evaluations of EONS within 2-hours post-delivery [8]. EONS categorization in NICU was determined by neonatologists unaware of the PCR and sequencing results. “Clinical” EONS was defined as the presence of “presumed” and/or “confirmed” EONS at ≤72 hours after birth. Clinical symptoms consistent with EONS included apnea, temperature instability, hypotension, bradycardia, lethargy, respiratory distress, and shock [23]. EONS was “confirmed” when cultures of neonatal blood or other biological fluid (urine, cerebrospinal fluid) were positive. The diagnosis of EONS was “presumed” in the presence of clinical symptoms and at least two of the following hematological criteria: absolute neutrophil count of <7,500 or >14,500 cells/mm^3^; absolute band count >1,500 cells/mm^3^; immature/total neutrophil (I:T) ratio >0.16; platelet count <150,000 cells/mm^3^ as previously described [12], [14], [24], [25], [26], [27]. All newborns with “confirmed” and/or “presumed” EONS received intravenous antibiotics. None of the term neonates (**Group 5)** displayed signs or symptoms of sepsis and were all discharged home at 48–72 hours of life.

### Af And Cb Collection And Clinical Tests Of Iai

AF was retrieved under sterile conditions by trans-abdominal amniocentesis (**Groups 1–4**) or at the time of Cesarean delivery after direct visualization of the intact amniotic bag through the uterine incision (**Group 5**). AF of **Groups 1–4** was sent to YNHH biochemical and microbiological laboratories for determination of glucose concentration, lactate dehydrogenase (LDH) activity, white (WBC) and red (RBC) blood cell counts and traditional Gram stain as rapid indicators to rule-out or confirm IAI. Results of these tests were used for clinical antepartum management as described above. A glucose level of ≤15 mg/dL [28], LDH activity ≥419 U/L [29], WBC count ≥30 cells/µL [27], [30], and/or positive Gram stain were considered indicative of intra-amniotic inflammation and/or infection. In prior studies we found that among these tests, the abnormal LDH activity was the best rapid biochemical indicator of intra-amniotic inflammation [27]. Microbial culturing was performed as described below. Excess AF was transported to the research laboratory where 1–2 mL were rapidly aliquoted into certified sterile DNA and DNAase-free tubes (Fisher Scientific, Inc. Pittsburgh, PA) for PCR analysis. The remaining volume was centrifuged at 1,000 g, 4°C for 15 min, and stored at −80°C for additional research analyses.

Umbilical vein CB was obtained immediately after delivery under sterile conditions and placed in serum and sodium citrate plasma tubes. Following collection, CB was centrifuged at 1,000 g at 4°C for 10 min. Serum, plasma and buffy coat were aliquoted in sterile DNA and DNAase-free tubes and stored at −80°C.

### Microbial Analysis By Culture

AF was cultured for aerobic and anaerobic bacteria, *Ureaplasma* and *Mycoplasma* species. The laboratory techniques used for isolation and identification of cultivable bacteria have been previously described [7]. The following media were used: Chocolate, Martin Lewis, MacConkey, Azido benzoic acid, Thioglycollate, *Bacteroides* Bile Esculin/Laked Blood Kanamycin Vancomycin (BBE/LKV) and Columbia CNA agar, *Ureaplasma* broth and agar. To identify anaerobic bacteria the biological specimens were cultured at 37°C in an anaerobic chamber (Forma Anaerobic System, Thermo Electron Co, Waltham, MA). After 5 days of culturing, the results of the microbiological tests were reported as final. Morphology of the bacterial colonies, medium reaction, Gram stain and the VITEK 2 automated card system (bioMérieux, Hazelwood, MO, http://www.biomerieux-usa.com) were used for bacterial identification. PCR was employed for separation among *Ureaplasma* species. For neonatal blood cultures, as per YNHH NICU protocol, 1 mL of neonatal blood was collected in aseptically from 2 separate sites within 2 hours of birth, prior to antibiotic treatment. Detection of early neonatal bloodstream infections was performed using an automated instrument (Bactec 9240, Becton Dickinson Diagnostics Instrument Systems, Sparks, MD, USA), which detects viable microorganisms within pediatric blood culture bottles (BD BATEC, Becton, Dickinson and Co, Sparks, MD).

### Bacterial Detection In Af And Cb By Pcr And Clone Analysis

DNA was extracted from 1 ml AF or CB serum as previously described [6]. Universal primers A17F (GTTTGATCCTGGCTCAG) and 1512R (TACCTTGTTACGACTT) were used for PCR amplification of the 16S rRNA genes as previously described [7]. These primers were modified from the well-known universal primers “27F” and “1492R” [31]. In A17F, we deleted the first three nucleotides from the 5′ end (AGA) in 27F; in 1512R, the first five nucleotides from the 5′ end of 1492R (ACGGC) were deleted. These deletions eliminated mismatches with sequences from members of *Panctomycetales, Thermotoga, Fervidobacterium* and some spirochetes [32]. As such, these two primers have improved homology to the conserved regions of 16S rRNA and can detect most eubacteria [33]. Using these primers and our working strain *F. nucleatum* 12230 as a testing strain, the limit of detection has improved from 1×10^6^ CFU reported in our previous study [6] to 1×10^4^ CFU (data not shown). As expected, not all species were detected with the same sensitivity. For example, the limit of detection for *Gardnerella vaginalis* was 1×10^6^ CFU, possibly due to two mismatches near the 5′ end in A17F (data not shown). All PCR products were observed by 1% agarose gel electrophoresis. Negative controls were included with each PCR reaction set to control for environmental or human contamination.

To identify the species amplified by PCR and to ensure that the PCR amplicons were indeed bacterial rRNA genes rather than artifacts, the PCR products were cloned into the pCR8 vector (Invitrogen, Carlsbad, CA). From each PCR positive sample, a total of 10–20 random clones were selected for full-length 16S rRNA gene sequence analysis (Genomics Core Facility, Case Western Reserve University, Cleveland, OH) using universal primers M13 forward (GTAAAACGACGGCCAGTG) and M13 reverse (GGAAACAGCTATGACCATG). The sequences were assembled and aligned using VectorNTI (Invitrogen). A local alignment algorithm (BLAST) was used against NCBI GenBank and Human Oral Microbiome Database (HOMD; http://www.homd.org) to assign each sequence to a species based on the generally accepted >97% sequence similarity with the reference strain [34]. Each PCR positive case was catalogued for the number (mono versus polymicrobial infections) and type of species. The sequences of the identified 16S rRNA genes are deposited in GenBank. Given severe limitations related to blood volume collection in premature babies, neonatal blood was not available for PCR analysis. This biological specimen was used for clinical purposes alone.

### Statistical Analyses

The Shapiro-Wilk test was used for data normality testing. Data is presented as mean and SEM or median and interquartile range as proper for normally or non-normally distributed data, respectively. Statistical analyses were performed with SigmaStat 11.0 (Systat Software Chicago, IL) software. Kruskal-Wallis ANOVA on Ranks followed by Dunn’s tests (to adjust for multiple comparisons) or Student t-tests were used as appropriate. Comparisons between proportions were done with Chi-square or Fisher’s exact tests. For each common species identified in AF and CB from the same pregnancy, Cohen’s Kappa coefficient was used to assess the co-occurrence, which was classified into the following categories: excellent (Kappa >0.8), good (0.6–0.8), moderate (0.4–0.6), fair (0.2–0.4), low (0–0.2), or none (0 or below). A *P* value of <0.05 was considered to indicate statistical significance.

## Results

### Clinical Characteristics Of Preterm Birth Cases

The demographic, clinical and outcome characteristics of pregnancies resulting in PTB (**Groups 1–4**) are presented in Table 1. **Group 5** was not considered for this comparative analysis on the basis of the normal pregnancy course with term delivery and the *a priori* designation of the samples as technical controls. **Groups 1** and **2** had lower GA at amniocentesis and at delivery, and delivered neonates with lower birth weights. The amniocentesis-to-delivery interval of women with IAI (**Group 2**) was shorter. The results of the clinical laboratory tests used for case management are presented in Table 2. Among the 6 cases with confirmed EONS (**Group 1**), 5 neonates were delivered in the setting of a positive AF culture result. An advanced degree of histological chorioamnionitis was seen for **Groups 1** and **2**. Higher degrees of neutropenia (abnormally low number of neutrophils) were observed for **Group 1** neonates.

**Table 1:** Demographical, clinical and outcome characteristics of preterm birth cases.

**Variables**	**Group 1**	**Group 2**	**Group 3**	**Group 4**	***P* value**
	**Culture-positive EONS (n = 6)**	**Presumed EONS & Yes IAI (n = 16)**	**Presumed EONS & No IAI (n = 7)**	**No EONS & No IAI (n = 7)**	
***Maternal demographical***					
Maternal age, *years* *Non-Caucasian race †Nulliparity †	30 [21]–[36]4 (67)3 (50)	24 [19]–[28]12 (75)7 (44)	24 [20]–[29]3 (43)1 (14)	32 [28]–[35]4 (57)3 (43)	0.1700.5020.513
***Maternal clinical***					
GA at amniocentesis, *wks* *Clinical chorioamnionitis †Antenatal steroids †Tocolytics †Antenatal antibiotics †PPROM †Amniocentesis-delivery, *hours* *Cesarean delivery †GA at delivery, *weeks* *	25 [24]–[26]0 (0)6 (100)3 (50)5 (83)3 (50)23 [4–60]4 (67)25 [25]–[26]	26 [24]–[30]0 (0)16 (100)9 (56)15 (94)9 (56)5 [4]–[14]3 (19)27 [25]–[29]	31 [31]–[32]1 (10)7 (100)4 (57)6 (86)4 (57)21 [5–53]1 (14)32 [31]–[33]	32 [30]–[33]0 (0)6 (86)5 (71)7 (100)6 (86)28 [12–155]3 (43)32 [31]–[34]	0.0020.6840.2350.5880.6600.5130.0290.106<0.001
***Pregnancy outcome***					
Birthweight, *grams* *1 minute Apgar <7 †5 minute Apgar <7 †	755 [678–933]5 (83)3 (50)	938 [740–1,571]9 (56)3 (19)	1790[1,535–2,275]2 (29)1 (14)	1840[1,645–2,145]3 (43)0 (0)	<0.0010.2360.145

* Data presented as median [interquartile range] and analyzed by Kruskal-Wallis ANOVA.

† Data presented as n (%) and analyzed by Chi square.

Abbreviations: EONS, early-onset neonatal sepsis; IAI, intra-amniotic infection; PPROM, preterm premature rupture of membranes.

**Table 2:** Results of clinical laboratory tests and placental pathology for preterm birth cases.

**Variables**	**Group 1**	**Group 2**	**Group 3**	**Group 4**	***P* value**
	**Culture-positive EONS (n = 6)**	**Presumed EONS & Yes IAI (n = 16)**	**Presumed EONS & No IAI (n = 7)**	**No EONS & No IAI (n = 7)**	
***Amniotic fluid***					
Glucose ≤15 mg/dL†LDH activity ≥419 IU/L†WBC count ≥30 cells/µL†Positive Gram stain †Positive cultures †	3 (50)4 (67)4 (67)4 (67)5 (83)	16 (100)16 (100)16 (100)13 (81)16 (100)	4 (57)2 (29)2 (29)0 (0)0 (0)	0 (0)0 (0)0 (0)0 (0)0 (0)	<0.001<0.001<0.001<0.001<0.001
***Placental pathology***					
HCA, *stages II or III* †Funisitis, *grades 1–4* †	6 (100)3 (50)	15 (94)11 (69)	5 (71)4 (57)	1 (14)0 (0)	<0.001<0.001
***Neonatal hematology***					
ANC, *cells/mL* *ABC, *cells/mL* *I:T ratio, *%* *Platelets, *cells/mLx1,000* *	489 [114–4,201]343[0–1256]10 [0–22]179 [171–267]	5,491 [2,086–8,632]2,876 [2456–4126]21 [14]–[30]232 [132–320]	2,592 [2,100–5,974]2,560 [82–4063]17 [1]–[20]209 [70–239]	3,388 [2,790–5,796]310[116–702]2 [2]–[8]217 [209–292]	0.106<0.0010.0080.615

* Data presented as median [interquartile range] and analyzed by Kruskal-Wallis ANOVA.

† Data presented as n (%) and analyzed by Chi square.

Abbreviations: EONS, early-onset neonatal sepsis; IAI, intra-amniotic infection; LDH, lactate dehydrogenase; WBC, white blood cell; HCA, histological chorioamnionitis; ANC, absolute neutrophil count; ABC, absolute band count; I:T ratio, ratio of immature-to-total neutrophils.

### Microbial Species Detected In Af

**Figure 1:**
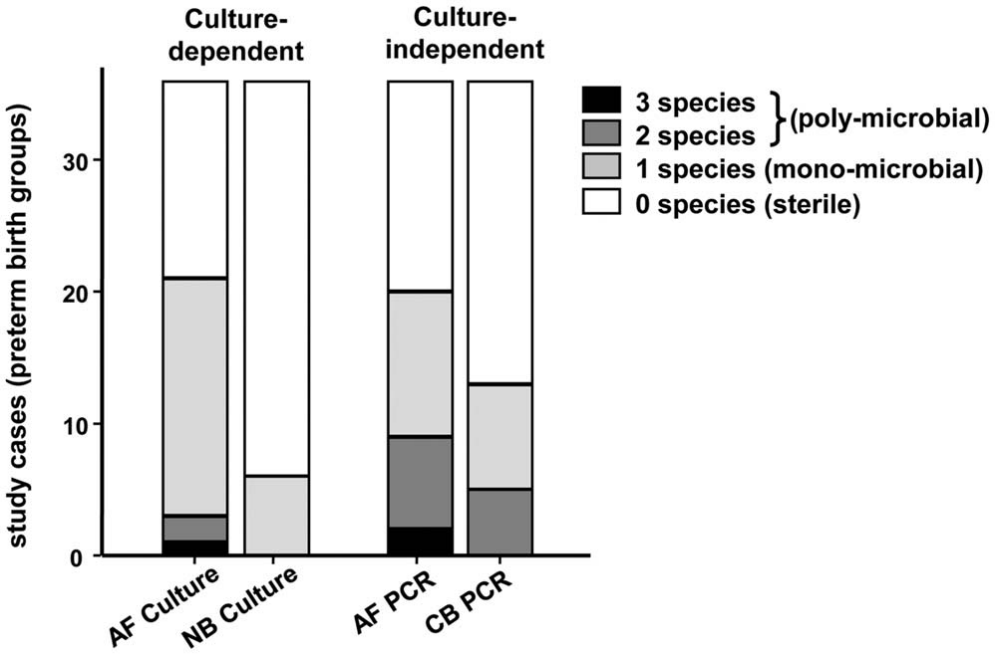
Proportion of cases found either sterile (open portion of the bar), mono- or poly-microbial (shaded portions of the bars). From left to right, the 4 compartments are: amniotic fluid (AF) and neonatal blood (NB) analyzed by culture, followed by AF and cord blood (CB) analyzed by the culture-independent methods of PCR and clone analysis.

A catalogue of all species identified by either culture-dependent or culture-independent methods is presented in Table 3. The accession numbers are listed in Table 4. All species identified shared >98% sequence similarity with the reference strains. In AF, culture-dependent methods identified bacteria in 21/36 (58%) of preterm samples which included 5/6 (83%) cases in **Group 1** and all 16 (70%) cases in **Group 2** (Table 5). Among the culture positive cases, 18/21 (86%) were mono-microbial (Table 3, Figure 1A). In the remaining poly-microbial AF infections, 2 cases each had two species (case 2-09 and case 2–10, Table 3) and one case (2-01) had 3 species (Figure 1A). The total 25 species in AF by traditional cultures, consolidated into 16 unique species (Table 5). No bacteria were detected in Groups 3 or 4 by culture.

**Table 3:** Bacterial species identified in matching amniotic fluid, cord and neonatal blood samples by culture and/or culture-independent methods.*.

**Group -Case**	**Culture-dependent**		**Culture-independent**	
	**Amniotic fluid**	**Neonatal blood**	**Amniotic fluid**	**Cord blood**
**1-01**	***Escherichia coli***–	***Escherichia coli***–	***Escherichia coli***–	***Escherichia coli*** *Streptococcus agalactiae*
**1-02**	–	*Streptococcus agalactiae*	–	*Escherichia coli*
**1-03**	***Escherichia coli***	***Escherichia coli***	***Escherichia coli***	–
**1-04**	***Streptococcus agalactiae***–	***Streptococcus agalactiae***–	***Streptococcus agalactiae*** ***Escherichia coli***	***Streptococcus agalactiae*** ***Escherichia coli***
**1-05**	*Mycoplasma*	*Staphylococcus* coagulase-neg.	*Mycoplasma hominis*	–
**1-06**	*Lactobacillus*	*Escherichia coli*	–	–
**2-01**	*Prevotella* *Ureaplasma* *Klebsiella pneumoniae*	–––	*Prevotella bivia*––	–––
**2-02**	***Fusobacterium nucleatum***–	––	***Fusobacterium nucleatum*** *Lachnospiraceae* sp.	***Fusobacterium nucleatum***–
**2-03**	***Ureaplasma***–	––	***Mycoplasma hominis*** ***Ureaplasma parvum***	***Mycoplasma hominis*** ***Ureaplasma parvum***
**2-04**	***Escherichia coli***–	––	***Escherichia coli*** *Ureaplasma parvum*	***Escherichia coli*** *Fusobacterium nucleatum*
**2-05**	*Peptostreptococcus asaccharolyticus*	–	*Peptoniphilus harei*	–
**2-06**	*Bacteroides stercoris*–	––	*Sneathia sanguinegens* *Peptoniphilus harei*	––
**2-07**	*Ureaplasma*	–	*Ureaplasma parvum*	–
**2-08**	*Streptococcus pneumoniae*	–	*Streptococcus pneumoniae*	–
**2-09**	*Ureaplasma* *Streptococcus anginosus*	––	*Ureaplasma parvum*–	––
**2-10**	***Fusobacterium nucleatum*** *Mycoplasma*	–**–**	***Fusobacterium nucleatum*** *Sneathia sanguinegens*	***Fusobacterium nucleatum***–
**2-11**	***Fusobacterium nucleatum***	**–**	***Fusobacterium nucleatum***	***Fusobacterium nucleatum***
**2-12**	–*Bacteroides ureolyticus*–	**–** **–** **–**	***Escherichia coli*** *Bacteroides ureolyticus* *Bergeyella sp.*	***Escherichia coli***––
**2-13** **2-13**	––*Streptococcus* viridans	–––	***Bergeyella sp.*** ***Fusobacterium nucleatum*** *Streptococcus mitis*	***Bergeyella sp.*** ***Fusobacterium nucleatum***–
**2-14**	*Listeria monocytogenes*	–	*Listeria monocytogenes*	–
**2-15**	*Haemophilus influenzae*	–	*Haemophilus influenzae*	–
**2-16**	***Ureaplasma***–	––	***Ureaplasma parvum*** *Prevotella bivia*	***Ureaplasma parvum***–
**3-01**	–	–	–	–
**3-02**	–	–	–	–
**3-03**	–	–	–	–
**3-04**	–	–	–	–
**3-05**	–	–	–	–
**3-06**	–	–	–	*Sneathia sanguinegens*
**3-07**	–	–	–	*Sneathia sanguinegens*

* Results are shown for Groups 1 and 2. No bacterial DNA was detected in any samples in Groups 3 and 4. The species identified in more than one compartment are shown in **bold**.

**Table 4:** Accession numbers of 16S rRNA genes detected in matching amniotic fluid and cord blood samples by PCR and clone analysis.

**Group-Case**	**Amniotic fluid**	**Accession number**	**Cord blood**	**Accession number**
1-01	*Escherichia coli*–	JN546076–	*Escherichia coli* *Streptococcus agalactiae*	JN546089JQ901486
1-02	–	–	*Escherichia coli*	JQ901487
1-03	*Escherichia coli*	JQ901469	–	–
1-04	*Streptococcus agalactiae* *Escherichia coli*	JN546078JN546077	*Streptococcus agalactiae* *Escherichia coli*	JN546091JN546090
1-05	*Mycoplasma hominis*	JN673565	–	–
2-01	*Prevotella bivia*	JQ901470	–	–
2-02	*Fusobacterium nucleatum* *Lachnospiraceae sp.*	JN546079JQ901471	*Fusobacterium nucleatum*–	JN546092–
2-03	*Mycoplasma hominis* *Ureaplasma parvum*	JN546081JN546080	*Mycoplasma hominis* *Ureaplasma parvum*	JN546094JN546093
2-04	*Escherichia coli* *Ureaplasma parvum*	JN546082JQ901472	*Escherichia coli* *Fusobacterium nucleatum*	JN546095JQ901488
2-05	*Peptoniphilus harei*	JQ901473	–	–
2-06	*Sneathia sanguinegens* *Peptoniphilus harei*	JQ901474JQ901475	––	––
2-07	*Ureaplasma parvum*	JQ901476	–	–
2-08	*Streptococcus pneumoniae*	JQ901477	–	–
2-09	*Ureaplasma parvum*	JQ901478	–	–
2-10	*Fusobacterium nucleatum* * *Fusobacterium nucleatum* * *Sneathia sanguinegens*	JN546083JN584646JQ901479	*Fusobacterium nucleatum*––	JN546096––
2-11	*Fusobacterium nucleatum*	JN546084	*Fusobacterium nucleatum*	JN546097
2-12	*Escherichia coli* *Bacteroides ureolyticus* *Bergeyella* sp.	JN546085JQ901480JQ901481	*Escherichia coli*––	JN546098––
2-13	*Bergeyella* sp.*Fusobacterium nucleatum* *Streptococcus mitis*	JN546086JN546087JQ901482	*Bergeyella* sp.*Fusobacterium nucleatum*–	JN546099JN546100–
2-14	*Listeria monocytogenes*	JQ901483	–	
2-15	*Haemophilus influenzae*	JQ901484	–	
2-16	*Ureaplasma parvum* *Prevotella bivia*	JN546088JQ901485	*Ureaplasma parvum*–	JN546101–
3-06	–	–	*Sneathia sanguinegens*	JQ901489
3-07	–	–	*Sneathia sanguinegens*	JQ901490

* Two subspecies of Fusobacterium nucleatum were identified. JN546083, F. nucleatum sbsp animalis; JN584646, F. nucleatum sbsp polymorphum.

**Table 5:** Summary of total number of species detected in amniotic fluid and cord blood by culture-dependent and independent methods.

Technique	Culture-dependent		Culture-independent	
Compartment	Amniotic fluid	Neonatal blood	Amniotic fluid	Cord blood
***Quantitative parameters***				
Positive cases *Negative cases *	21 (58)15 (42)	6 (17)30 (83)	20 (56)16 (44)	13 (36)23 (64)
Species detected †	25	6	31	18
Unique species detected †	16	3	15	7

* Data presented as n (%).

† Data presented as n.

Using culture-independent methods, bacterial species were detected in 20/36 (56%) AF samples, all in **Groups 1** and **2** (Tables 3 and 5). In all these cases, bacteria were also detected by culture. However, compared to the traditional cultures, more poly-microbial infections were identified by PCR (Fisher’s exact *P* = 0.043), with 11 cases (55%) mono-microbial (Table 3 and Figure 1A), 7 cases (35%) with two species, and 2 cases (10%) with 3 species. A total of 31 species among the preterm AF samples were identified by the culture-independent methods, which converged to 15 unique species (Tables 3 and 5). No bacterial DNA was detected in Groups 3, 4, or 5.

Combining the culture and culture-independent methods, the microbial species detected in AF were (with decreasing prevalence): *Ureaplasma parvum* and *Escherichia coli* (n = 5 each), *Fusobacterium nucleatum* (n = 4), *Bergeyella sp., Peptoniphilus indolicus*, and *Sneathia sanguinegens* (n = 2 each), and *Bacteroides stercoris, Bacteroides ureolyticus, Haemophilus influenzae, Klebsiella pneumonia, Lachnospiraceae sp., Listeria monocytogenes, Mycoplasma hominis, Prevotella bivia*, *Streptococcus agalactiae* (GBS), *Streptococcus anginosus, Streptococcus mitis, and Streptococcus pneumoniae* (n = 1 each) (Table 6).

**Table 6:** Alphabetical list of bacterial species identified by culture and culture-independent methods.

Bacterial species	# of Cases in AF	# of Cases in CB	Kappa coefficient***
*Bacteroides stercoris* *	1	–	–
*Bacteroides ureolyticus*	1	–	–
*Bergeyella sp* **	2	1	0.65
*Escherichia coli*	5	5	1.00
*Fusobacterium nulceatum*	4	5	0.87
*Haemophilus influenzae*	1	–	–
*Klebsiella pneumoniae* *	1	–	–
*Lachnospiraceae* sp. **	1	–	–
*Listeria monocytogenes*	1	–	–
*Mycoplasma hominis*	1	1	1.00
*Peptoniphilus harei* **	2	–	–
*Prevotella bivia*	1	–	–
*Sneathia sanguinegens* **	2	2	-0.06
*Streptococcus agalactiae*	1	2	0.65
*Streptococcus anginosus* *	1	–	–
*Streptococcus mitis*	1	–	–
*Streptococcus pneumoniae*	1	–	–
*Ureaplasma parvum*	5	2	0.53

* identified only by culture.

** identified only by culture-independent methods.

*** Kappa coefficient is calculated for species identified in the paired AF-CB samples.

### Microbial Species In Nb And Cb

**Figure 2:**
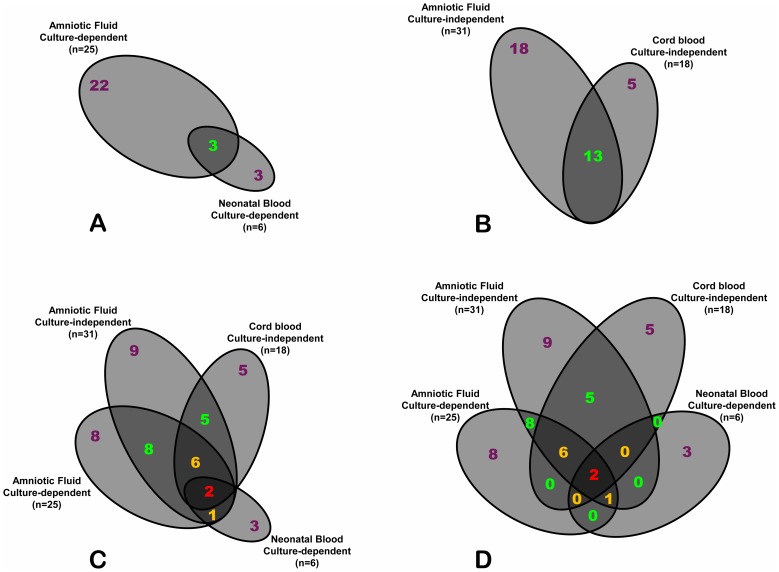
Euler-Venn diagrams showing the number of species shared among compartments of each case (co-occurrences). Compartments of each case were analyzed by either the culture-dependent (**A**) or culture-independent methods (**B**). The size of each space (ellipse) is proportional to the number of species in each compartment. 4-way intersecting diagrams of the 4 compartments showing either non-null spaces that are proportional to the number of species (Euler-like diagram) (**C**) or the complete non-scaled design which includes spaces with no overlapping species (Venn diagram) (**D**).

By study design, neonatal blood cultures were positive only in **Group 1** (Table 3). All 6 cases were mono-microbial (Figure 1A) with only 3 unique species reported in neonatal blood (i.e. *Escherichia coli, GBS and Staphylococcus*, Table 3). In CB, culture-independent techniques identified bacterial DNA in 13/36 (36%) of preterm cases, more than double the number identified by traditional cultures in neonatal blood (*P*<0.001). The PCR-positive CB cases were identified in **Groups 1** (3/6) (confirmed EONS), **2** (8/16) (presumed EONS with positive IAI), and **3** (2/8) (presumed EONS with negative IAI), but not in Groups 4 or 5 (no EONS). Eight out of thirteen (62%) of the PCR-positive CB cases were mono-microbial (Table 3 and Figure 1A) and 2 species were reported in the remaining 5/13 (38%) cases. None of the CB samples had 3 or more species identified. The total number of 18 species found in CB by PCR consolidated into 7 unique species: *E. coli* and *F. nucleatum* (n = 5 each), *S. sanguinegens*, GBS, *U. parvum* (n = 2 each), *Bergeyella* sp. and *M. hominis* (n = 1 each) (Table 5).

### Overlap Between The Af And Cb Microbiomes

To investigate the level of non-random association between bacterial species encountered in AF and CB, we performed an overlap analysis. In Figure 2A we present Euler-Venn diagrams illustrating that culture dependent-methods (considered as clinical gold standard) were able to find only 3 unique species overlapping between neonatal blood and AF of the same case. When the analysis was shifted to the results obtained using culture-independent methods, the number of species shared between paired CB and AF increased to 13 (Figure 2B). With the exception of GBS in case 1-01, *E. coli* in case 1-02, *F. nucleatum* in case 2-04, and *S. sanguinegens* in cases 3-06 and 3-07, the majority of the species (13/18, 72%) identified in CB was also detected in AF (Tables 3 and 6). Figure 2C illustrates the Euler diagram of non-null intersections of all 4 compartments with the size of the areas proportional to the number of species shared or unique to each compartment. Only 2 species overlapped across all compartments of the same pregnancy (Table 3, case 1-01: *E. coli* and case 1-04: GBS) when both culture-dependent and independent results were taken into consideration. As several possible intersections among biological compartments lacked shared species, the complete 4-way Venn diagram with the number of species for each possible intersection is shown in Figure 2D.

Based on the Kappa coefficient, the species exhibiting the best agreement between AF and CB include *E. coli, F. nucleatum,* and *M. hominis* (Kappa score >0.8, excellent agreement), followed by *Bergeyella* and GBS (Kappa score 0.6-0.8, good agreement), and *U. parvum* (Kappa score 0.4–0.6, moderate agreement) (Table 6). No agreement was observed for *S. sanguinegens*, because it was identified in non-matching AF and CB. The 16 rRNA genes of the common species identified in paired AF and CB shared 99.9–100% sequence identity, with no more than two nucleotide mismatches (Table 7). The limited mismatch could be due to errors in PCR and/or sequencing. No identical sequences were found between different cases (data not shown).

**Table 7:** Similarity of 16S rRNA gene sequences of the 13 common species shared between AF and CB from the same pregnancy.

Group–Case	Amniotic fluid species	Cord blood species	% Identity*
1-01	*Escherichia coli*	*Escherichia coli*	100% (1497/1497)
1-04	*Streptococcus agalactiae*	*Streptococcus agalactiae*	100% (1504/1504)
	*Escherichia coli*	*Escherichia coli*	99.9% (1495/1497)
2-02	*Fusobacterium nucleatum*	*Fusobacterium nucleatum*	99.9% (1472/1474)
2-03	*Mycoplasma hominis*	*Mycoplasma hominis*	100% (1473/1473)
	*Ureaplasma parvum*	*Ureaplasma parvum*	99.9% (1471/1472)
2-04	*Escherichia coli*	*Escherichia coli*	99.9% (1477/1478)
2-10	*Fusobacterium nucleatum*	*Fusobacterium nucleatum*	99.9% (1472/1474)
2-11	*Fusobacterium nucleatum*	*Fusobacterium nucleatum*	100% (1474/1474)
2-12	*Escherichia coli*	*Escherichia coli*	99.9% (1495/1497)
2-13	*Bergeyella* sp	*Bergeyella* sp	100% (1482/1482)
	*Fusobacterium nucleatum*	*Fusobacterium nucleatum*	99.9% (1473/1474)
2-16	*Ureaplasma parvum*	*Ureaplasma parvum*	99.9% (1471/1472)

* Expressed in percent identity with number of identical bases/total bases in alignment shown in parentheses.

### Analysis Of Discrepancies Between Species Identified By Culture-Dependent And Culture-Independent Methods

In AF, the culture-dependent method reported 7 species which were not confirmed by PCR: Lactobacillus, Klebsiella pneumoniae, Ureaplasma, Mycoplasma, Streptococcus anginosus, Peptostreptococcus asaccharolyticus, and Bacteroides stercoris (Table 3). For Peptostreptococcus asaccharolyticus, clone analysis identified a closely related species, Peptoniphilus harei (Table 3, cases 2-05). Conversely, PCR identified 14 AF species that were not found by cultures. Among these, P. harei, S. sanguinegens, Bergeyella sp., and Lachnospiraceae sp. were identified only by PCR (Table 3). Streptococcus mitis identified by PCR in case 2–13 is a member of Streptococcus viridans, consistent with the findings by culture. Seventeen species were identified in the same specimen by both methods, including: U. parvum (n = 4), E. coli (n = 3), F. nucleatum (n = 3), B. ureolyticus (n = 1), H. influenza, L. monocytogenes, M. hominis, P. bivia, GBS and S. pneumoniae (1 case each).

In the group of newborns with positive blood cultures, out of the total 6 species cultivated from neonatal blood, 4 were not identified by PCR: E. coli (n = 2), Streptococcus agalactiae (n = 1) and Staphylococcus coagulase-negative (n = 1). However, among all samples of CB in this study, PCR identified 16 species that were not found by cultures. Of these, F. nucleatum (n = 5), S. sanguinegens and U. parvum (n = 2 each), Bergeyella sp. and M. hominis (n = 1 each) were detected in CB only by culture-independent methods.

## Discussion

There is a paucity of metagenomic studies in human pregnancy, and in particular in fetal and neonatal septic conditions related to IAI. In this study we evaluated for the first time bacterial diversity in AF and CB in parallel by using culture-dependent and independent techniques. Culture-dependent methods more often suggested a single-species profile in AF or neonatal blood. Conversely, by 16S-rRNA sequencing, most AF infections were found to be poly-microbial with 15 distinctive species identified as potential etiologic agents of IAI. The same technique indicated that the majority of CB infections were, in contrast, mono-microbial. Only 7 distinct species were identified in CB as potential etiologic agents of EONS. Collectively, these results suggest that the microbial diversity of AF and CB differ with respect to specific bacterial species and with the detection method. The reduced microbial diversity observed in CB compared to AF, both by culture and by PCR, indicates that only a limited number of species could invade into the fetal compartment. Alternatively, this discrepancy could be due to administration of antibiotics before and/or during labor as AF was collected prior to the use of antibiotics while CB was collected after delivery.

Using culture-independent methods, we previously identified significantly more microbial species in AF than the traditional culturing methods [7]. Prior to our studies [6], [7], *Bergeyella* sp., an uncultivated oral species, had never been associated with pregnancy complications. In the current study, *Bergeyella* was once again identified by the culture-independent methods, further validating the involvement of this uncultivated species in IAI. A novel discovery of the current research was identification of two new species, *Lachnospiraceae sp.,* and *P. harei*, in AF. Neither had been linked to IAI before. We anticipate that many more species will be discovered to be involved in pregnancy complications using the culture-independent technologies.

The culture-dependent and independent methods complement, but cannot entirely replace, each other. As reported in our previous study [7], a few AF species were detected by traditional cultures but not by PCR. Possible technical explanations were degradation of bacterial DNA prior to PCR analysis and the limited number of clones analyzed in each sample. Alternatively, bacterial culture results could have been prone to errors. The identity of cultivated species in AF reported by the microbiology laboratory provides some insight. For example, *Klebsiella pneumoniae* (Table 3, case 2-01) has been reported to occur from cross-contamination of the culture analyzer [35]. Contamination may also be the likely scenario for case 1-05 with coagulase-negative *Staphylococcu*s [36]. In one miss-matched case a closely related species was identified by culture-independent techniques (*Peptoniphilus asaccharolyticus* vs. *Peptoniphilus harei*). These disagreements are likely due to taxonomical or typing errors due to limitations of the culturing method. *P. asaccharolyticus* (formerly known as *Peptostreptococcus asaccharolyticus*) is a Gram positive anaerobic coccus known to have the same phenotypical features as *P. harei* and its identity is often mistaken by cultures [37]. Neither *P. harei* nor *Sneathia spp* are included on the VITEK2 card.

Using conventional culturing methods, only 3 commons species are shared between AF and neonatal blood. When the analysis was performed on paired AF-CB samples using culture-independent methods, the number was significantly increased to 13 species. This suggests that fetal exposure in the setting of IAI may be incomplete based on microbial cultures alone. The high sequence-similarity levels (99.9-100%) among the 13 species shared between AF and CB argue against randomness as reason for their co-occurrence.

Our understanding of the extent and identity of the AF pathogens that gain access to the fetal compartment is incomplete. The use of metagenomics techniques allowed us to appreciate for the first time the microbial differences and similarities between AF and CB as two distinct yet unquestionably linked compartments. Our results suggest that only some bacteria, i.e. *Bergeyella sp., E. coli*, *F. nucleatum, M. hominis,* GBS and *U. parvum*, are more likely to breech the fetal barrier than others. In particular, *F. nucleatum*, *E. coli* and *M. hominis* emerged as the most “transmissible” species of IAI and hence of EONS-I based on their Kappa scores. In comparison, *U. parvum*, one of the most prevalent species detected in AF, was only detected in 2 of 5 matching CB samples. *Bergeyella* was detected in one of the 2 matching CB samples, while no agreement between AF and CB was observed for *S. sanguinegens*. Interestingly, *F. nucleatum* and GBS were detected in more cases of CB than AF. These results indicate that not all infections in CB are related to those in AF.

The finding of *F. nucleatum*, a common oral anaerobe, as one of the most prevalent etiological agents of EONS-I is novel. While *F. nucleatum* has been frequently detected in IAI [7], [38], [39], its association with EONS has never been reported before. The high frequency of fetal translocation of *F. nucleatum* may be due to its ability to invade epithelial and endothelial cells [40], [41]. We have shown that *F. nucleatum* colonizes the mouse placenta by crossing the endothelium, followed by induction of Toll-like receptor 4-mediated inflammatory responses leading to fetal loss [41], [42]. Placental colonization by *F. nucleatum* requires FadA, a filamentous adhesin exposed on the surface of the bacteria [43], [44], [45]. FadA binds to the cell-junction molecule, vascular endothelial (VE)-cadherin, causing it to migrate away from the cell-cell junction, thus increasing the endothelial permeability [46]. This is likely the mechanism utilized by *F. nucleatum* to cross the placental barrier and to breach into the fetal compartment. Impairment of the endothelial permeability by FadA also allows the penetration by other bacterial species, such as *E. coli* [46]. This explains why *F. nucleatum* is often found in mixed infections. The species co-identified with *F. nucleatum* in this study, such as *Bergeyella sp., Lachnospiraceae, S. sanguinegens,* and *S. mitis,* are all common oral species [47]. It is possible that they co-migrate with *F. nucleatum* from the oral cavity. This observation is consistent with our previous report of co-translocation of diverse oral species from the dental plaque and saliva to the murine placentas [48].

One major limitation of the study is the small sample size of the groups, which reflect the real-life situation in any NICU in the world, where the number of culture-positive cases of EONS are rare to detect, despite best practice in sample collection and blood culture. In the absence of a positive culture, the duration of treatment of the babies becomes dependent upon subjective clinical criteria and hematological criteria-based scoring systems. The hematological criteria used in the present manuscript have been validated in multiple previous studies [12], [14], [24], [25], [26], [27]. While not ideal, they offer us some guidance in making clinical decisions, and the grouping of the cases is clinically-relevant to any practicing neonatologist. The grouping and sample sizes were primarily driven by the minimum number of culture positive cases (n = 6) that we felt was required in a pilot study of this nature. Having a positive or negative IAI helps the neonatologist in making clinical decisions. In addition, this grouping allowed us to have somewhat similar sample sizes for comparative purposes.

Culture “confirmed” or “presumed” EONS are a frequent encounter in pregnancies complicated by IAI [49], [50]. The findings presented above stress the value of molecular technologies in establishing comprehensive and quantitative measures of bacterial translocation between AF and the fetal compartment. As shown, culturing of neonatal blood did not identify bacteria in any of the newborns with “presumed” EONS (**Groups 2** and **3)**. In the current study the neonatal blood was not available for PCR analysis, and CB was used instead. Several possible scenarios can explain why bacterial DNA was detected in CB by PCR in a significant portion (10/23; 43%), although not in all newborns with “presumed” EONS. Similar to AF, a significant limitation of culture is the small number of microbiological cultivation protocols restricted to a few known pathogens [51]. This may explain the inability to detect previously unrecognized uncultivated or difficult-to-cultivate species such as *Bergeyella sp.*, *F. nucleatum*, and *Leptotrichia* (*Sneathia*). Bacterial killing by appropriate immune defense mechanisms and/or use of antenatal antibiotics represent additional explanations given that microbial cultures will only identify viable and multiplying species whereas PCR may amplify DNA of both live and dead bacteria. Presence of bacterial DNA in CB of 43% of newborns with presumed EONS is of significant clinical relevance because even if the identified species are no longer viable, such microbes may still represent etiologic agents of the EONS syndrome (EONS-II variant). Bacterial PAMPs, including free DNA of dead bacteria, can actively engage fetal Toll-like receptors initiating an inflammatory cascade in neonates with “presumed” EONS [52]. However, the significantly lower diversity of CB compared to AF reported in this study argues against simple passive leakage of PAMPs and favors the argument that some (but not all) bacterial species actively invade the fetus in a manner consistent with EONS-I pathogenic variant. The remaining cases of EONS where bacteria was not identified by either culture or PCR were likely either EONS-II (triggered by passage of DAMPs) or EONS-III (passage of cytokines) variants.

### Conclusions

This study demonstrates the following: (1) the profile of etiologic agents responsible for EONS is greater than currently appreciated by culture; (2) bacteria in AF are more likely than others to breech the fetal barrier, underscoring IAI as a possible ascendant of EONS; and (3) among the newly identified etiologic agents of EONS-I, our study places *F. nucleatum* at the same level on the importance scale with *E. coli*. A better understanding of IAI and of EONS variants from etiologic and pathogenic standpoints is critical for development of targeted treatments to improve neonatal outcome.
